# Insufficient Vitamin C Levels among Adults in the United States: Results from the NHANES Surveys, 2003–2006

**DOI:** 10.3390/nu13113910

**Published:** 2021-10-30

**Authors:** Jennifer Crook, Ann Horgas, Saun-Joo Yoon, Oliver Grundmann, Versie Johnson-Mallard

**Affiliations:** 1 Center for Health Equity and Community Engagement Research, Mayo Clinic Hospital, Jacksonville, FL 32224, USA; 2 Biobehavioral Nursing Science, College of Nursing, University of Florida, Gainesville, FL 32610, USA; ahorgas@ufl.edu (A.H.); yoon@ufl.edu (S.-J.Y.); 3 Entrepreneurial Programs in Medicinal Chemistry, College of Pharmacy, University of Florida, Gainesville, FL 32610, USA; grundman@ufl.edu; 4 Dean, College of Nursing, Kent State University, Kent, OH 44240, USA; VJOHNS29@kent.edu

**Keywords:** ascorbate, Vitamin C insufficiency, public health, food insecurity, nutrition

## Abstract

Vitamin C, well-established in immune function and a key factor in epigenetic inflammatory modifications, is only obtained through consistent dietary intake. Identifying individuals at risk for Vitamin C insufficiency may guide prevention and treatment, however, national surveillance has not been evaluated in the United States since 2006. A descriptive, cross-sectional secondary analysis was performed utilizing data from the 2003–2006 National Health and Nutrition Examination Surveys (NHANES) assessing non-institutionalized adults. Five categories of plasma Vitamin C were delineated: deficiency (<11 μmol/L), hypovitaminosis (11–23 μmol/L), inadequate (23–49 μmol/L), adequate (50–69 μmol/L), and saturating (≥70 μmol/L). Results indicated 41.8% of the population possessed insufficient levels (deficiency, hypovitaminosis, and inadequate) of Vitamin C. Males, adults aged 20–59, Black and Mexican Americans, smokers, individuals with increased BMI, middle and high poverty to income ratio and food insecurity were significantly associated with insufficient Vitamin C plasma levels. Plasma Vitamin C levels reveal a large proportion of the population still at risk for inflammatory driven disease with little to no symptoms of Vitamin C hypovitaminosis. Recognition and regulation of the health impact of Vitamin C support the goal of Nutrition and Healthy Eating as part of the Healthy People 2030.

## 1. Introduction

Prior to the Coronavirus-19 (COVID-19) pandemic, the United States (USA) led the world in the incidence of food insecurity among high-income nations [1,2], with as many as 40% of households affected [3]. Although it is not clear yet how recent restrictions such as school closures and quarantines have affected geographic locations of food insecurity, and despite research identifying insufficiencies of Vitamin C to be a global issue with many countries affected [4,5,6,7], nutritional surveillance is not often part of routine screenings during clinic visits or admittance to inpatient settings. Vitamin C is emerging as a potential benefit in the prevention and treatment of COVID-19 infections [8,9,10] and other inflammatory conditions [11,12,13,14,15,16]; though the underlying plasma Vitamin C status of individuals in the USA is currently not well-known. There exists an emergent need to identify subclinical vitamin C insufficiencies (<23 μmol/L) because higher supplementation may be needed in patients with Vitamin C hypovitaminosis to achieve the saturated levels necessary for alleviating inflammation states and supporting immune system defenses [11].

Vitamin C is a water-soluble vitamin that cannot be synthesized in the human body and is usually included in a well-balanced diet from fruits and vegetables such as strawberries, kiwis, peppers, broccoli, cauliflower, kale, pineapples, and citrus fruits, among others. Plasma concentration of Vitamin C is tightly regulated through intestinal absorption, tissue transport, and renal reabsorption [17] and is the second most reliable gauge of circulating Vitamin C after leukocytic measurement. Vitamin C is a well-known antioxidant possessing multiple beneficial properties. Although a comprehensive list of its full benefits has still not been clearly identified, many of its valuable properties are believed to be the result of its ability to act as an electron donor [18,19,20]. These include neutralizing free radical oxygen species, inhibition of low-density lipoprotein (LDL) oxidation [21], alleviating chronic inflammation [12], supporting growth and development of healthy gut microbiota [9], enhancing neutrophil motility [10,11], aiding the proliferation of natural killer cells [12], recycling Vitamin E [22,23], and enhancing the bioavailability of iron [24]. The benefits of Vitamin C in specific epigenetic inflammatory pathways, including the ability to act as a cofactor to both start and complete DNA and histone demethylations, is being realized [25,26,27,28]. Some countries are identifying the need to adjust recommend intakes based on optimal health instead of the previously defined deficiency prevention. Current recommendations for daily intake of Vitamin C are inconsistent worldwide with ranges from 40 to 220 mg/day [29] and there remains little to no consistent evaluation of population intake or standard guidelines for increased consumption/supplementation when necessary.

Nationwide monitoring of plasma Vitamin C status has not been recorded in the USA since 2006, in which 13% of the population was found to be deficient (<10.99 μmol/L) [30]. However, Vitamin C insufficiencies (including not only deficiency but hypovitaminosis and inadequate plasma levels) may partly explain the increased incidence of chronic inflammation common to numerous chronic diseases as well as highlight potential hindrances in immune system defense. There are multiple known Vitamin C-lowering components including stress [31,32], poverty [4,33], obesity [34,35], and smoking [36,37,38]. Coupled with Western low nutritionally dense, the need for plasma Vitamin C assessment in routine screenings as well as admission to inpatient settings becomes more apparent. There is much evidence supporting increased inflammation and disease with Vitamin C deficiency and hypovitaminosis, but research is identifying greater immune benefit at saturating levels [14,39], higher than currently defined adequate levels of vitamin C (50–69 μmol/L). There is also little to no current assessment of the range between hypovitaminosis and adequate (labeled inadequate in this study) or how this range, with no signs or symptoms of Vitamin C deficiency, relates to increased risk of inflammation and disease. The examination of the prevalence of all five categories of Vitamin C: deficiency, hypovitaminosis, inadequate, adequate, and saturating levels may benefit clinicians in prevention and treatment, rationale for supplementation during increased need, and guidance of public health policy for funding for federal dietary programs such as Food Stamps, National School Lunch Program, and Women, Infant, and Children (WIC). This study meets the goal of Nutrition and Healthy Eating as part of the Healthy People 2030 [40] to describe the prevalence of plasma levels of Vitamin C in the population of the USA based on five defined categories: deficiency (<11 μmol/L), hypovitaminosis (11–23 μmol/L), inadequate (23–49 μmol/L), adequate (50–69 μmol/L), and saturating (≥70 μmol/L).

## 2. Materials and Methods

This study is a secondary data analysis of the National Health and Nutrition Examination Survey (NHANES; 2003–2006) sponsored by the National Center for Health Statistics (NCHS), the Centers for Disease Control and Prevention (CDC). NHANES employed a multi-step randomization approach for nationally representative sampling of a non-institutionalized population, requiring sample weights to be calculated and used in analysis. Certain populations are over-sampled and sample weights are calculated so that demographics closely align with current national census information. Interviews were conducted in homes and physical examinations with laboratory tests were done in mobile examination clinic (MEC) visits. A four-year sample weight was calculated from the specific coding instructions listed on the CDC website regarding combining the 2003–2006 surveys [30]. All participants provided informed consent and all identifying information removed prior to survey datasets being made publicly available online.

**Figure 1:**
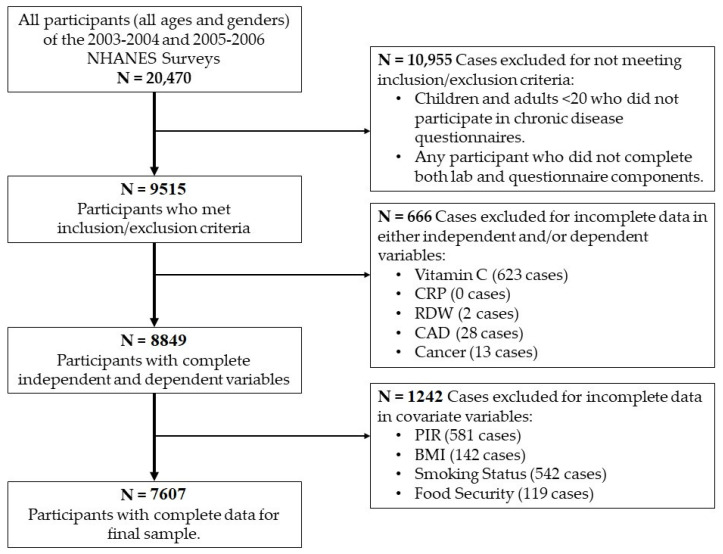
Sample Selection Flowsheet.

Inclusion criteria consisted of both sexes and all ethnicities, ages ≥20 years of age, of non-institutionalized civilian participants, who were able to give informed consent, and participated in both questionnaire and laboratory measurements and retained complete information in all study variables. Excluded from analysis were children and participants with missing data in combined participant laboratory and questionnaire. Selection of sample participants for inclusion is identified in Figure 1. From the NHANES completed and published for the years 2003–2004 and 2005–2006, there were a total of 20,470 participants who participated. After application of the inclusion and exclusion criteria, a total of 7607 participants were included in the final sample. The study was approved by the University of Florida Institutional Review Board (IRB# 201902929).

The measures, including variable names and recoding justifications are included as follows:

Sex: Sex information listed in the Demographic Data Set where respondents answered “male” or “female.”

Age: Age information was also included in the Demographic Questionnaire. Respondents answered in a range of values, and individuals aged >85 years were top-coded at 85. As the NHANES had grouped portions of the participants by top-coding removing the ability to define this variable differently, this study recoded the age variable into three categorical variables included in analysis: Young Adult, Middle Adult, and Late Adult.

Body Mass Index (BMI): BMI measurements were listed in Exam Data. Upon initial analysis of examining outliers, one participant was excluded for an abnormally high BMI. Further examination of the respondent’s listed height and weight revealed that the BMI listed was an input error and was correctly calculated, and participant included for analysis.

Food Security: Food security information was included in the Food Security Questionnaire. Respondents answered “yes” or “no” to the question, “Are you worried you will run out of food?”

Smoking Status: Smoking status was included in the Smoking Recent Tobacco Use Questionnaire. Respondents answered “yes” or “no” to the question, “Used tobacco/nicotine in the last 5 days?”

Vitamin C: Vitamin C (ascorbic acid) in serum was measured by isocratic high-performance liquid chromatography with electrochemical detection at 650 mV1. Peak area quantitation was based on a standard curve generated from three different concentrations of an external standard. The measured vitamin C was converted from mg/dL to μmol/L by multiplying by 56.78. NHANES maintains quality assurance and quality control protocols that meet 1988 Clinical Laboratory Improvement Act mandates. A full description of the specimen collection, laboratory processing method, and quality control procedures can be found on the NHANES website. Initial analysis identified correlations between the continuous Vitamin C variable to the other tested variables. To analyze the prevalence across five ordinal strata of plasma Vitamin C categories, the Vitamin C variable was recoded and grouped according to the following categories: deficiency (0–10.99 μmol/L), hypovitaminosis (11–23.99 μmol/L), inadequate (24–49.99 μmol/L), adequate (50–69.99 μmol/L), and saturating (≥70 μmol/L) based on participant plasma levels.

Ethnicity: Ethnicity information was collected in the Demographic Questionnaire. Respondents chose from the following categories: Mexican American, Other Hispanic, Non-Hispanic White, Non-Hispanic Black, or Other.

Poverty to Income Ratio (PIR): Poverty to Income Ratio information was collected in the Demographic Questionnaire. NHANES calculated PIR using Department of Health and Human Services poverty guidelines to determine qualification for financial assistance for federal aid programs such as Food Stamps, the National School Lunch Program [23]. The variable was recoded into categorical values of: High PIR (participants below 30% of the poverty line eligible for government assistance, or ≤$25,000 per year), Medium PIR (considered middle class with a household income of $25–$75,000 per year), and Low PIR (considered high-income earners with household incomes of >$75,000 per year).

Vitamin C Intake: Total nutrient intakes were captured in the Dietary Data Questionnaire dataset utilizing the What We Eat In America Questionnaire developed by the USA Department of Agriculture and USA Department of Health and Human Services. Participants recalled all foods and beverages eaten for two nonconsecutive days (the first interview done in the mobile exam clinic and the second conducted over the phone 3–10 days later). To identify the specific nutrients included in the intake data, the USDA Food and Nutrient Database for Dietary Studies, 2.0 (FNDDS 2.0) was utilized. Vitamin C intake was recorded as a range of values in milligrams. A full description of the quality control measures employed in obtaining dietary data may be found on the NHANES website.

Descriptive statistics were used to analyze the prevalence of plasma Vitamin C in the population of the USA Data from the 2003–2006 NHANES datasets downloaded in statistical analysis system (SAS) transport file format and Statistical Analysis System (SAS) version 9.4 (SAS Institute Inc., Cary, NC, USA). SAS files were converted to Statistical Package for the Social Sciences (SPSS) (IBM SPSS Statistics for Windows, Version 26.0. Armonk, NY, USA) for analysis. Four-year sample weights were calculated per the National Center for Health Statistics (NCHS) recommendations. A separate NHANES weighting variable was provided for researchers to analyze dietary data results as participants were more likely to have study visits that occurred on the weekend and food intake is different on weekdays versus weekends. Since two days’ worth of dietary intake was evaluated in this study, the appropriate sample weights were utilized as instructed on the NHANES website. Pearson’s chi-square tests were utilized for categorical variables and analysis of variance (ANOVA) testing was employed for continuous variables. The continuous variables of BMI, plasma Vitamin C, and Vitamin C intake were assessed for normality and found to be positively skewed, although with large sample sizes, violations of normality do not noticeably affect results and thus do not require transformations [41]. BMI and Vitamin C intakes were assessed for homogeneity of variances and found the assumption violated; thus, a Welch correction was applied. In all statistical tests, a *p*-value of less than 0.05 was considered statistically significant. Means with Standard Deviations (S.D.s), percentages, and frequencies were used to present continuous variables, and proportions were used for categorical variables.

## 3. Results

A total of 7607 unique cases were utilized in this study. A description of the weighted sample characteristics is shown in Table 1. The sample was comprised of 40.1% adults between 40–59 years of age, 51.3% females, and 73.6% Non-Hispanic Whites. Most participants were non-smokers (70.6%) and did not worry about running out of food (85.9%). A significant percentage (63.8%) of the sample was categorized as possessing high PIR levels. Mean BMI was 28.68 kg/m^2^ (SD 6.44) and mean plasma Vitamin C level 54.63 μmol/L (SD 28.62).

### 3.1. Prevalence of Vitamin C Levels

**Figure 2:**
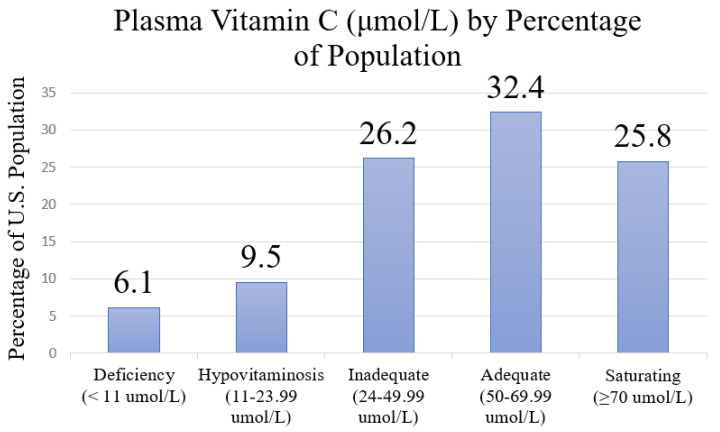
Prevalence of Plasma Vitamin C Levels in the USA Population in 2003–2006.

The prevalence of plasma Vitamin C levels in the population of the USA is delineated by five quartiles: deficiency, hypovitaminosis, inadequate, adequate, and saturating (see Figure 2). More than half of the participants (58.2%) had sufficient levels of plasma Vitamin C, composed of 32.4% with adequate and 25.8% with saturating plasma Vitamin C levels. However, 41.8% of the sample had insufficient levels of Vitamin C. Specifically, 6.1% of the participants exhibited deficient levels, 9.5% had hypovitaminosis levels, and 26.2% had inadequate levels.

### 3.2. Associations between Sample Characteristics and Vitamin C Levels

Table 2 demonstrates the associations between sample characteristics across the five plasma Vitamin C quintiles. Significant relationships were identified for each variable.

Males (*n* = 3699, 48.7%) were significantly more likely to exhibit insufficient levels of plasma Vitamin C (as categorized in the deficiency, hypovitaminosis, and inadequate quintiles) compared to females (3908, 51.3%) [χ2 = 215.82, df = 3.42, *p* < 0.001]. Assessment of saturating levels of Vitamin C revealed a wide disparity between males (19.3%) and females (32.3%).

There were statistically significant age differences in Vitamin C categories [χ2 = 250.45, df = 5.74, *p* <0.001]. Participants in the young (*n* = 2751, 36.5%) and middle adulthood (*n* = 2295, 40.1%) groups were more likely to have inadequate plasma Vitamin C levels. Those in the late adulthood category (*n* = 2561, 22.3%) had the highest percentage of saturating plasma Vitamin C levels.

Ethnicity exhibited significant differences among plasma Vitamin C categories [χ2 = 148.28, df = 8.6, *p* = <0.001]. Non-Hispanic Blacks (*n* = 1536, 10.5%) demonstrated the highest levels of insufficient plasma Vitamin C (combined deficiency, hypovitaminosis, and inadequate quintiles) with 48.5%, followed by ‘Other’ (*n* = 290, 4.9%) with 44.7%, and Mexican Americans (*n* = 1516, 7.6%) with 43.8%. Participants of the category ‘Other Hispanics’ (*n* = 230, 3.4%) revealed the lowest percentage of insufficient Vitamin C levels (combined deficiency, hypovitaminosis, and adequate quintiles) with 40.8%, followed by Non-Hispanic Whites (41.4%). White participants displayed the highest levels of both deficiency (7.6%) and saturating (28.2%) plasma Vitamin C.

Participants who reported high Poverty to Income Ratios (PIR) (*n* = 5206, 63.8%) and medium PIR (*n* = 1614, 22.7%) possessed equal levels of insufficient Vitamin C with 43.9% and 43.5%, respectively. Participants who reported low PIR (*n* = 787, 13.5%), indicating higher levels of income, had the lowest percentage of insufficient levels of plasma Vitamin C with 33.6% [χ2 = 80.47, df = 4.93, *p* = 0.002]. Medium PIR displayed the highest proportion of individuals with Vitamin C deficiency (9.5%).

Smoking status also found significance across the categories of plasma Vitamin C [χ2 = 673.25, df = 3.34, *p* = <0.001]. Smokers (*n* = 1997, 29.4%) were more likely to fall within the insufficient plasma Vitamin C quintiles of deficiency, hypovitaminosis, and inadequate (60.8%) than non-smokers (*n* = 5610, 70.6%), who revealed only 34.8%. Only 14.7% of smokers reached saturating levels of Vitamin C compared to non-smokers (30.7%).

Participants who acknowledged food insecurity (*n* = 1449, 14.1%) had higher percentages of insufficient Vitamin C levels (deficiency, hypovitaminosis, and inadequate) compared to those who denied food insecurity (*n* = 6158, 85.9%) [χ2 = 114.59, df = 3.29, *p* = <0.001] with food insecure participants exposing 56.5% compared to 40.1% of those not food insecure. Additionally, a wide gap in saturating Vitamin C levels between participants with food insecurity (16.2%) and those without food insecurity (27.6%).

Mean BMIs indicated significant differences across the categories of plasma Vitamin C [F = 58.65, df = 4, *p* = <0.001]. Participants with insufficient plasma Vitamin C levels (deficiency, hypovitaminosis, and inadequate categories) possessed ranges of BMI from 29.0 kg/m^2^–29.8 kg/m^2^, while participants with sufficient Vitamin C (adequate and saturating categories) showed BMI ranges of 27.1 kg/m^2^–28.6 kg/m^2^.

Vitamin C intake also showed significant differences across the categories of plasma Vitamin C for both non-consecutive days [Day One intake: F = 134.43, df = 4, *p* ≤ 0.001; Day Two intake: F = 88.84, df = 4, *p* < 0.001]. Mean ranges of reported dietary intake of Vitamin C were 39.4 mg to 124.9 mg for the two days collected.

## 4. Discussion

These findings are consistent with the literature that examined baseline plasma Vitamin C levels prior to implementation of Vitamin C intervention [42] and suggest that insufficient Vitamin C levels remain a current public health issue.

Categorizing participants into five strata of plasma Vitamin C demonstrates the most impacted group by inadequate dietary intake and surveillance. Males were significantly more likely to be in insufficient levels of Vitamin C than females. This finding is consistent among studies examining gender differences across multiple countries [5,43,44]. Although some reasons may be dietary differences and volumetric dilution due to higher muscle mass [45], more research is needed to identify clear causality. Minorities were disproportionately affected by insufficiencies of Vitamin C, with both Non-Hispanic Black and Mexican Americans comprising the largest percentages of inadequate plasma Vitamin C as well as the lowest rates of saturating Vitamin C levels. This study corroborates the limited findings regarding Vitamin C plasma status among Black and White ethnicities [46,47]. More recent research has identified the Asian ethnicity to be at higher risk of decreased Vitamin C [6], though not captured in this study. While traditional dietary practices may partly explain the incidence, other attributes considered are the increased risk of stress in minority populations [48], higher incidence of food insecurity [49], and higher rates of obesity and type II diabetes [50], of which all are associated with decreased Vitamin C. PIR confirmed that low socioeconomic status (SES) is a crucial factor in decreased Vitamin C levels. Yet, participants, who were categorized as medium PIR (those who would be considered middle class and not eligible for federal food assistance programs), held similar rates of insufficient plasma Vitamin C levels as those in the lower income category and held the highest proportion of participants with Vitamin C deficiency. It provides evidence of a possibly overlooked segment of the population with nutritional insufficiencies contributing to increased inflammation. This study corroborated previous findings regarding the effect of smoking on decreased plasma Vitamin C levels due to the development of oxidants and in vivo oxidative stress [4,51,52] from smoking. Individuals with food insecurity also displayed a significantly higher percentage of insufficient levels of Vitamin C. Although there is limited information regarding the nutritional deficiencies specific to areas considered food deserts, some studies have identified decreased reported dietary intake of Vitamin C among the food insecure [53], though residents are at increased risk of chronic inflammation [54]. BMI mean differences across plasma Vitamin C levels to a small degree substantiates evidence of an increased risk of insufficient Vitamin C levels in individuals with increased BMI possibly due to increased rates of inflammation [55,56], volumetric dilution due to decreased muscle mass [45], and/or poor dietary intake [7]. Comparing participant dietary intakes and the small effect size seen when compared to plasma Vitamin C identifies the disparity between reported intake and readily available Vitamin C within the body. This also underscores the importance of assessment of plasma/leukocytic Vitamin C status and not relying solely on dietary intake data.

This study has limitations. The use of secondary data limits the ability to define or propose new variables to the dataset. Categorizing the age variable in this study identified significant differences in plasma Vitamin C though a further delineation into age ranges including later adulthood (young-old and old-old) should be considered. Vitamin C plasma levels were last observed in the NHANES studies in 2006. More recent data on the national plasma Vitamin C data would provide more updated information. Food security issues remain unclear under the current pervasiveness and severity related to unemployment and lockdowns brought on by the coronavirus pandemic [57]. The USDA updated definitions of food security in 2020, introduced in 2006, so variables included in this study are not the most current [58]. The findings cannot be generalized to institutionalized adults, such as residents of long-term care facilities. Finally, the ethnicity variables were not as diverse as today, so this study cannot clearly distinguish ethnic differences in Vitamin C levels.

Research efforts are suggested to identify the prevalence and risks of insufficient plasma Vitamin C levels in individuals of all ages (including infants and children) and more diverse race/ethnicities. Pregnancy status is another area where research is advised, as needs may transiently increase during the progression of the pregnancy [59,60,61]. It is also recommended to investigate both low SES and those considered middle class, as this study identified both to be significant predictors for decreased Vitamin C. Further examination of Vitamin C insufficiencies in older adults is suggested as findings may not be applied to residents of long-term care facilities, a sector with known nutritional inadequacies that would also benefit from increased surveillance and treatment [62,63,64].

Since plasma concentration of Vitamin C is tightly regulated [17] and is second to leukocytic measurement as a reliable estimate of actual body storage, it is recommended that future research study laboratory processing advancements for Vitamin C so it can be assessed in both inpatient and outpatient settings.

## 5. Conclusions

The identification of increased supplementation necessary to reverse hypovitaminosis [11] as well as the uncertainty of the pervasiveness of food insecurity caused by the coronavirus pandemic, highlight the importance of nutritional surveillance and better tailored interventions. Research has previously identified that approximately 25% of the population consumed less than the recommended daily amount (RDA) of Vitamin C [2]. This study provides evidence from plasma blood levels that suggest that percentage of Vitamin C insufficiency is much higher. Disregarding nutritional insufficiencies leads not only to a lack of awareness and policy change within the community but limits potentially beneficial treatment options. The vitamin C status of the population. This study provides a clear examination of the prevalence of insufficient Vitamin C by separating participant plasma levels into quintiles of deficiency, hypovitaminosis, inadequate, adequate, and saturating. A large segment of the sample contained participants with inadequate levels of Vitamin C, exposing a population who may not exhibit any overt symptoms of scurvy, but are still at risk for inflammatory driven diagnoses, allowing practitioners to redefine patients who are considered at risk. Vitamin C has a long history in research, though its benefits and mechanisms of action are still not fully understood. Surveillance is not consistent, and this study contributes to a limited amount of literature delineating the prevalence of five specific categories of plasma Vitamin C levels within the USA The increased prevalence of individuals with insufficient plasma Vitamin C provides conclusive evidence that dietary intake of Vitamin C is still not where it should be.

## Figures and Tables

**Table 1:** Vitamin C Prevalence Sample Description (N = 7607).

Characteristics	*n*	Weighted *n* (%)	Mean (*S.D.*)	Range
Gender				
Male	3699	48.7% ± 0.7%		
Female	3908	51.3% ± 0.7%		
Age				
Young Adult 20–39	2751	37.5% ± 0.7%		
Middle Adult 40–59	2295	40.1% ± 0.7%		
Late Adult ≥60	2561	22.4% ± 0.5%		
Race/Ethnicity				
Mexican American	1516	7.6% ± 0.2%		
Other Hispanic	230	3.4% ± 0.3%		
Non-Hispanic White	4035	73.6% ± 0.5%		
Non-Hispanic Black	1536	10.5% ± 0.3%		
Other	290	4.9% ± 0.3%		
Family PIR ^1^				
High (0–1.5)	5206	63.9% ± 0.5%		
Medium (1.51–4.5)	1614	22.6% ± 0.5%		
Low (>4.51)	787	13.5% ± 0.5%		
Smoking Status				
Yes	1997	29.4% ± 0.6%		
No	5610	70.6% ± 0.6%		
Food Insecure				
Yes	1449	14.1% ± 0.4%		
No	6158	85.9% ± 0.4%		
BMI ^2^	7607		28.68 (6.44)	13.36–76.07
Plasma Vitamin C ^3^	7607		54.37 (28.62)	0.6–274.20
Vitamin C Intake Day 1 ^4^	7468		91.91 (104.14)	0.0–2261.00
Vitamin C Intake Day 2 ^4^	6886		95.30 (99.31)	0.0–1308.40

^1^ Poverty to Income Ratio; ^2^ Body Mass Index (kg/m^2^); ^3^ Plasma Vitamin C (μmol/L); ^4^ Vitamin C Intake (mg).

**Table 2:** Sample Characteristics by Plasma Vitamin C Quintiles.

Characteristics	*n* (Weighted %)	Deficiency	Hypo-Vitaminosis	Inadequate	Adequate	Saturating	*p*-Value	
		*n* = 467,	*n* = 722	*n* = 1991, 26.17%	*n* = 2467, 32.43%	*n* = 1960, 25.77%		Effect Size ^5^
		6.14%	9.49%					
Gender								
Male	3699 (48.7% ± 0.5)	8.6% (±0.8)	12.4% (±0.8)	28.2% (±1.2)	31.6% (±1.1)	19.3% (±0.8)	<0.001	0.17
Female	3908 (51.3% ± 0.5)	4.9% (±0.6)	8.2% (±0.7)	23.0% (±1.0)	31.7% (±1.3)	32.3% (±1.3)		
Adulthood Stage								
Young (20–39)	2751 (36.5% ± 0.8)	6.6% (±0.8)	11.0% (±0.9)	28.9% (±1.1)	31.8% (±1.3)	21.7% (±1.4)	<0.001	0.11
Middle (40–59)	2295 (40.1% ± 0.8)	7.9% (±1.0)	11.0% (±0.7)	26.3% (±1.0)	32.8% (±1.2)	22.1% (±1.2)		
Late (≥60)	2561 (22.3% ± 0.5)	4.6% (±0.6)	7.6% (±0.8)	18.5% (±0.8)	29.4% (±1.5)	40.0% (±1.1)		
Race/Ethnicity							<0.001	
Mexican American	1516 (7.6% ± 1.1)	3.8% (±0.8)	7.7% (±1.3)	32.3% (±1.5)	37.6% (±1.8)	18.7% (±1.5)		
Other Hispanic	230 (3.4% ± 0.5)	1.3% (±0.8)	10.5% (±3.1)	29.0% (±3.1)	38.6% (±4.3)	20.6% (±3.7)		0.08
Non-Hispanic White	4305 (73.6% ± 2.1)	7.6% (±0.7)	10.6% (±0.7)	23.2% (±0.9)	30.5% (±1.1)	28.2% (±1.1)		
Non-Hispanic Black	1536 (10.5% ± 1.2)	5.5% (±0.8)	8.3% (±1.0)	34.7% (±1.6)	32.3% (±1.7)	19.1% (±1.2)		
Other	290 (4.9% ± 0.4)	3.8% (±1.5)	12.6% (±2.5)	28.3% (±2.2)	33.5% (±2.6)	21.8% (±2.0)		
Family PIR ^1^							0.002	
High (0–1.5)	5206 (63.8% ± 1.1)	6.3% (±0.6)	11.1% (±0.7)	26.5% (±0.7)	31.5% (±1.2)			
Medium (1.51–4.5)	1614 (22.7% ± 0.5)	9.5% (±1.6)	9.8% (±1.1)	24.2% (±1.3)	31.0% (±1.6)	24.5% (±0.9)		0.06
Low (>4.51)	787 (13.5% ± 0.6)	3.7% (±1.1)	7.0% (±1.0)	22.9% (±2.4)	33.4% (±1.6)	25.5% (±1.8)		
						33.1% (±2.1)		
Smoking Status							0.001	
Yes	1997 (29.4% ± 1.0)	14.8% (±1.0)	17.0% (±1.2)	29.0% (±1.3)	24.6% (±1.5)	14.7% (±1.0)		0.28
No	5610 (70.6% ± 1.0)	3.3% (±0.4)	7.4% (±0.5)	24.1% (±0.8)	34.6% (±0.8)	30.7% (±0.9)		
Food Insecure							<0.001	
Yes	1449 (14.1% ± 0.8)	9.7% (±1.2)	13.9% (±1.5)	32.9% (±1.1)	27.3% (±1.7)	16.2% (±1.2)		0.12
No	6158 (85.9% ± 0.8)	6.2% (±0.6)	9.6% (±0.6)	24.3% (±0.8)	32.3% (±1.0)	27.6% (±0.9)		
BMI ^2^	7607 (100%)	29.0 (±7.3)	29.8 (±7.4)	29.8 (±6.8)	28.6 (±6.1)	27.1 (±5.5)	<0.001 ^4^	0.02
Vitamin C Intake ^3^							<0.001 ^4^	
Day One	7468 (98.1%)	39.4 (±55.0)	50.9 (±61.7)	73.5 (±94.1)	102.2 (±104.8)	124.9 (±119.5)		0.06
Day Two	6886 (90.5%)	41.6 (±68.7)	60.2 (±74.2)	84.2 (±89.1)	104.7 (±106.8)	118.8 (±103.4)		

^1^ Poverty to Income Ratio; ^2^ Body Mass Index (kg/m^2^) presented as mean/SD; ^3^ Reported as mg/day; ^4^ With Welch correction; ^5^ Cramer’s V (categorical) and R^2^ (continuous).

## Data Availability

Publicly available datasets were analyzed in this study. This data can be found on the Centers for Disease Control and Prevention (CDC) website at: https://www.cdc.gov/nchs/nhanes/index.htm.
